# HER2 as a potential biomarker of lymph node metastasis in undifferentiated early gastric cancer

**DOI:** 10.1038/s41598-020-61567-1

**Published:** 2020-03-24

**Authors:** Sanghoon Han, Sungjin Park, Jungsuk An, Jun-Young Yang, Jun-Won Chung, Yoon Jae Kim, Kyoung Oh Kim, Dong Kyun Park, Kwang An Kwon, Woon Kee Lee, Seungyoon Nam, Jung Ho Kim

**Affiliations:** 1 Department of Internal Medicine, Jeju National University School of Medicine, Jeju, Korea; 2 College of Medicine, Gachon University, Incheon, Republic of Korea; 3 Gachon Institute of Genome Medicine and Science, Gachon University Gil Medical Center, Incheon, Republic of Korea; 4 Department of Pathology, Gachon University Gil Medical Center, Gachon University School of Medicine, Incheon, Republic of Korea; 5 Department of Surgery, Gachon University Gil Medical Center, Gachon University School of Medicine, Incheon, Republic of Korea; 6 Department of Internal Medicine, Gachon University Gil Medical Center, Gachon University School of Medicine, Incheon, Republic of Korea; 7 Department of Life Sciences, Gachon University, Seongnam, Republic of Korea; 8 Gachon Advanced Institute of Health Sciences & Technology, Gachon University, Incheon, Republic of Korea; 9 Gachon Medical Research Institute, Gachon University Gil Medical Center, Incheon, Republic of Korea

**Keywords:** Gastric cancer, Surgical oncology

## Abstract

Human epidermal growth factor receptor 2 (HER2) is implicated in several cancers, including gastric cancer. However, limited data are available regarding its clinical significance in early gastric cancer (EGC). We evaluated the clinical significance of HER2 overexpression in patients with EGC. We retrospectively reviewed 727 patients who underwent surgical treatment for EGC between October 2010 and August 2017. HER2 expression was analysed in 680 EGC cases by immunohistochemistry and classified as negative (0 and 1+), equivocal (2+), or positive [overexpression (3+)]. Among patients with differentiated EGC, the number of patients with HER2 overexpression was not significantly different from that of HER2-negative patients in terms of age, sex, tumour size, location, gross type, depth of invasion, presence of lymphovascular invasion (LVI), and presence of lymph node metastasis (LNM). However, in patients with undifferentiated EGC, HER2 overexpression was significantly correlated with LVI and presence of LNM compared with HER2-negative patients. Multivariate analysis indicated HER2 overexpression as a good predictive marker of LNM in patients with undifferentiated EGC. HER2 expression is associated with LNM in undifferentiated EGC. Therefore, the importance of HER2 overexpression in EGC should not be overlooked, and further studies are needed to identify its clinical significance.

## Introduction

Gastric cancer is the fifth most common malignancy worldwide^1^. In Korea, gastric cancer is the second highest in men and fourth highest in women^2^. In Korea, adult males and females over 40 years old are being screened for gastric cancer through a national cancer screening program. This screening program recommends upper gastrointestinal (GI) endoscopy or upper GI imaging every 2 years. This screening has led to early treatment of gastric cancer and lower mortality^3,4^. Therefore, early diagnosis has become a very important social concern because of these advantages. In Korea and Japan, the detection rate in the early stage of gastric cancer has increased up to 70%^4–6^. As the detection rate of early gastric cancer (EGC) has increased, treatment and prognosis of EGC have become very important.

In clinical practice, the presence of lymph node metastasis (LNM) in EGC is very important for two reasons. First, the prognosis of EGC is highly related to LNM. The 10-year survival rates with and without LNM are 72% and 92%, respectively. Therefore, predicting the possibility of LNM in EGC is an important issue related to survival^7^. Second, LNM is the most valuable factor in determining EGC treatment modalities. Although conventional treatment for gastric cancer is surgery, endoscopic treatment is frequently performed for EGC treatment^8^. To perform endoscopic treatment, it is assumed that there is no LNM. Therefore, it is very important to confirm the presence of LNM in EGC before endoscopic resection, a non-invasive treatment.

In fact, the endoscopic treatment indications of EGC were determined to reflect the criteria for the absence of LNM in the surgical EGC^9,10^. In other words, since a prospective randomised study with statistical significance is difficult and unethical, the existing endoscopic resection criteria can be referred to as criterion based on the retrospective study using surgically resected tissues.

Recently, there have been reports that LNM is rarely found in the expanded criteria for endoscopic treatment of EGC. Based on this, endoscopic resection is frequently performed in patients with EGC classified by expanded criteria at many centres^11^. Expanded criteria have shown an effort to broaden the scope of endoscopic resection using histologic differentiation, size, presence of ulcers, and depth of submucosal invasion. To date, vague pathological and clinical factors have been mainly used^12–14^. Recently, there was a concern that use of expanded criteria may lead to a higher rate of LNM in EGC^13,15^. Therefore, attempts to predict the likelihood of LNM in EGC with only these factors could be incomplete. Additional parameters are needed to better predict the potential for LNM in EGC.

If we can generate a tumour biology-based prediction using molecular markers, we can more accurately predict LNM in EGC. Since molecular biology testing methods have recently been developed, it will be possible to use molecular biomarkers for EGC diagnosis. There have been many studies on various targets that can be practically applied in the diagnostic process^1,16^. If these markers are included in criteria to determine the indication for endoscopic treatment, then predicting LNM may be more accurate.

Recently, the presence of human epidermal growth factor receptor 2 (HER2; also known as ERBB2) expression in advanced gastric cancer (AGC) has played an important role in treatment^17^. HER2 is a member of the HER family tyrosine receptor kinase (RTKs) encoded by proto-oncogene ERBB2^18^. There are reports that HER2 plays an important role in cancer cell survival and aggressiveness^19,20^. In addition, it is known that ERBB2 gene mutation overexpression, leading to HER2, occurs early in carcinogenesis^21^.

Although there is no report of the exact role of HER2 in EGC, we hypothesised that HER2 may play an important role in the aggressiveness of EGC, based on these preliminary reports. We investigated the relationship between HER2 and EGC LNM under this background.

## Results

### Baseline characteristics of patients with EGC

**Figure 1:**
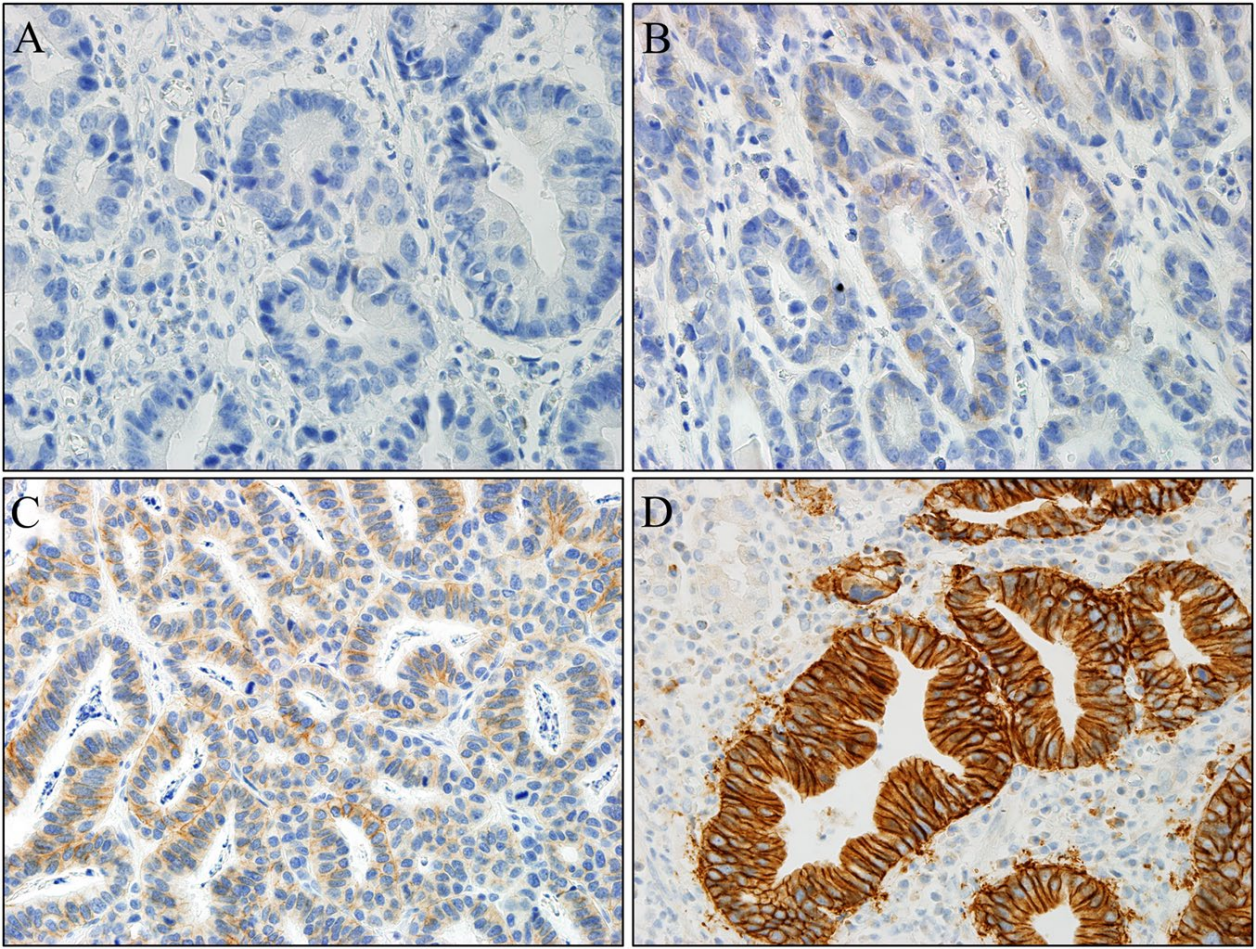
Representative cases showing an example of scoring in HER2 immunohistochemistry staining. (**A**) 0 Negative: No response to staining, no tumour cell membrane responds to staining at all. Cytoplasmic staining was not included in the scoring. (**B**) 1+ Negative: Tumour cells with very faint staining or barely recognisable staining. (**C**) 2+ Equivocal: refers to the case of weak or moderate complete staining on the basolateral and lateral sides of tumour cells. Tangentially cut cells in columnar cells tended to show a complete membrane staining pattern. (**D**) 3+ Positive: Tumour cells show a strong complete staining reaction in the basolateral and lateral membranes. It should be taken into account that some of the cells that appeared to show a complete membrane staining pattern were often tangentially cut columnar cells. Original magnification x 400 (**A**–**D**). HER2, Human epidermal growth factor receptor 2.

Baseline characteristics of the 680 patients are shown in Table 1. Of 680 patients, 456 (67.1%) were males, and the mean age was 59.9 ± 11.8 years. The mean tumour size was 32.3 ± 19.4 mm. The most common location was the lower third (299, 44.0%) and lesser curvature of the stomach (248, 36.5%) in the longitudinal and horizontal axis, respectively. The most common gross appearance was the depressed type (358, 52.6%). Mucosal cancer was similar to EGC with submucosal invasion in 343 patients (50.4%), with a similar ratio to EGC with submucosal invasion (49.6%). The undifferentiated-type EGC was slightly more prevalent than the differentiated-type EGC in 364 patients (53.5%). In total, 200 (29.4%), 262 (38.5%), 182 (26.8%), and 36 cases (5.3%) showed HER2 expression scores of 0, 1+, 2+, and 3+, respectively (Fig. 1). Further, 140 patients (20.6%) had lymphovascular invasion (LVI), and 80 (11.8%) had LNM.

**Table 1:** Baseline characteristics of patients with EGC.

	Total (n = 680)
Age	59.9 ± 11.8
**Sex**	
Male	456 (67.1%)
Female	224 (32.9%)
Tumour size	32.3 ± 19.4
**Vertical location**	
UT	145 (21.3%)
MT	236 (34.7%)
LT	299 (44.0%)
**Horizontal location**	
AW	149 (21.9%)
GC	107 (15.7%)
PW	176 (25.9%)
LC	248 (36.5%)
**Gross type**	
Elevated	109 (16.0%)
Flat	213 (31.3%)
Depressed	358 (52.6%)
**Depth of invasion**	
M	343 (50.4%)
SM	337 (49.6%)
**Histology**	
WD	76 (11.2%)
MD	240 (35.3%)
PD	132 (19.4%)
SRC	190 (27.9%)
Mixed	38 (5.6%)
Mucinous	2 (0.3%)
Lymphoid stroma	2(0.3%)
**Status of HER2**	
Negative (0)	200 (29.4%)
Negative (1+)	262 (38.5%)
Equivocal (2+)	182 (26.8%)
Positive (3+)	36 (5.3%)
**LVI**	
No	540 (79.4%)
Yes	140 (20.6%)
**LN metastasis**	
No	600 (88.2%)
Yes	80 (11.8%)

EGC, early gastric cancer; SMEGC, synchronous multiple EGC; UT, upper third; MT, mid third; LT, low third; AW, anterior wall; GC, great curvature; PW, posterior wall; LC, lesser curvature; LVI, lymphovascular invasion; VL, vertical location; HL, horizontal location.

### Clinicopathological characteristics according to HER2 protein expression intensity

The clinicopathological results according to the expression degree of HER2 protein using immunohistochemistry (IHC) are shown in Table 2. As the expression level of HER2 protein increased, the proportion of patients aged ≥ 60 years and males tended to increase significantly (Table 2). In addition, significant increasing trends were observed in the proportions of large tumours (>20 mm), submucosal cancer, cases with LVI, and differentiated-type EGC. Unlike this phenomenon, tumour location and gross appearance did not show any tendency dependent on the degree of HER2 protein expression.

**Table 2:** Clinicopathological characteristics according to HER2 protein expression intensity.

	Total (n = 680)	Negative (n = 462)	Borderline (n = 182)	Positive (n = 36)	p-value
Age					<0.001
<60	340 (50.0%)	252 (54.5%)	76 (41.8%)	12 (33.3%)	
≥60	340 (50.0%)	210 (45.5%)	106 (58.2%)	24 (66.7%)	
Sex					0.002
M	456 (67.1%)	291 (63.0%)	138 (75.8%)	27 (75.0%)	
F	224 (32.9%)	171 (37.0%)	44 (24.2%)	9 (25.0%)	
Tumour size					0.027
≤20	176 (25.9%)	132 (28.6%)	37 (20.3%)	7 (19.4%)	
>20	504 (74.1%)	330 (71.4%)	145 (79.7%)	29 (80.6%)	
Vertical location					0.928
UT/MT	381 (56.0%)	259 (56.1%)	101 (55.5%)	21 (58.3%)	
LT	299 (44.0%)	203 (43.9%)	81 (44.5%)	15 (41.7%)	
Gross appearance					0.065
E	109 (16.0%)	68 (14.7%)	31 (17.0%)	10 (27.8%)	
FD	571 (84.0%)	394 (85.3%)	151 (83.0%)	26 (72.2%)	
Depth of invasion					<0.001
M	343 (50.4%)	259 (56.1%)	70 (38.5%)	14 (38.9%)	
SM	337 (49.6%)	203 (43.9%)	112 (61.5%)	22 (61.1%)	
Histology					<0.001
Differentiated	316 (46.5%)	177 (38.3%)	110 (60.4%)	29 (80.6%)	
Undifferentiated	364 (53.5%)	285 (61.7%)	72 (39.6%)	7 (19.4%)	
LVI					0.017
No	540 (79.4%)	377 (81.6%)	139 (76.4%)	24 (66.7%)	
Yes	140 (20.6%)	85 (18.4%)	43 (23.6%)	12 (33.3%)	
Node metastasis					0.296
No	600 (88.2%)	410 (88.7%)	161 (88.5%)	29 (80.6%)	
Yes	80 (11.8%)	52 (11.3%)	21 (11.5%)	7 (19.4%)	

EGC, early gastric cancer; SMEGC, synchronous multiple EGC; UT, upper third; MT, mid third; LT, low third; AW, anterior wall; GC, great curvature; PW, posterior wall; LC, lesser curvature; LVI, lymphovascular invasion; VL, vertical location; HL, horizontal location.

### Clinicopathologic differences in HER2 expression by EGC differentiation

After excluding equivocal expression of the protein, subgroup analysis was performed to determine differences in the characteristics of patients with HER2 protein over-expression in comparison with those without HER2 protein expression (Tables 3 and 4). Among patients with differentiated EGC (316/680, 46.5%), those with HER2 overexpression (29/316, 9.2%) did not show clinically significant differences compared with HER2-negative patients (177/316, 56.0%) in terms of age, sex, tumour size, location, gross type, depth of invasion, presence of LVI, and presence of LNM.

**Table 3:** Differences according to the presence or absence of HER2 expression in differentiated-type EGC.

	Total (n = 206)	Negative (n = 177)	Positive (n = 29)	p-value
Age				0.633
<60	72 (35.0%)	63 (35.6%)	9 (31.0%)	
≥60	134 (65.0%)	114 (64.4%)	20 (69.0%)	
Sex				0.486
M	168 (81.6%)	143 (80.8%)	25 (86.2%)	
F	38 (18.4%)	34 (19.2%)	4 (13.8%)	
Tumour size				0.158
≤20	66 (32.0%)	60 (33.9%)	6 (20.7%)	
>20	140 (68.0%)	117 (66.1%)	23 (79.3%)	
Vertical location				0.145
UT/MT	102 (49.5%)	84 (47.5%)	18 (62.1%)	
LT	104 (50.5%)	93 (52.5%)	11 (37.9%)	
Gross type				0.615
E	56 (27.2%)	47 (26.6%)	9 (31.0%)	
F/D	150 (72.8%)	130 (73.4%)	20 (69.0%)	
Depth of invasion				0.219
SM	99 (48.1%)	82 (46.3%)	17 (58.6%)	
M	107 (51.9%)	95 (53.7%)	12 (41.4%)	
LVI				0.199
No	167 (81.1%)	146 (82.5%)	21 (72.4%)	
Yes	39 (18.9%)	31 (17.5%)	8 (27.6%)	
Node metastasis				>0.99
No	184 (89.3%)	158 (89.3%)	26 (89.7%)	
Yes	22 (10.7%)	19 (10.7%)	3 (10.3%)	

EGC, early gastric cancer; SMEGC, synchronous multiple EGC; UT, upper third; MT, mid third; LT, low third; AW, anterior wall; GC, great curvature; PW, posterior wall; LC, lesser curvature; LVI, lymphovascular invasion; VL, vertical location; HL, horizontal location.

**Table 4:** Clinicopathological significance of HER2 expression in undifferentiated-type EGC.

	Total (n = 292)	Negative (n = 285)	Positive (n = 7)	Univariate analysis	Multivariate analysis	
				p-value	OR (95% CI)	p-value
Age				0.236		NA
<60	192 (65.8%)	189 (66.3%)	3 (42.9%)		NA	
≥60	100 (34.2%)	96 (33.7%)	4 (57.1%)		NA	
Sex				0.271		NA
M	150 (51.4%)	148 (51.9%)	2 (28.6%)		NA	
F	142 (48.6%)	137 (48.1%)	5 (71.4%)		NA	
Tumour size				0.685		NA
≤20	73 (25.3%)	72 (25.3%)	1 (14.3%)		NA	
>20	219 (75.0%)	213 (74.7%)	6 (85.7%)		NA	
Vertical location				0.438		NA
UT/MT	178 (61.0%)	175 (61.4%)	3 (42.9%)		NA	
LT	114 (39.0%)	110 (38.6%)	4 (57.1%)		NA	
Gross type				0.426		NA
E	22 (7.5%)	21 (7.4%)	1 (14.3%)		NA	
F/D	270 (92.5%)	264 (92.6%)	6 (85.7%)		NA	
Depth of invasion				0.245		NA
M	166 (56.8%)	164 (57.5%)	2 (28,6%)		NA	
SM	126 (43.2%)	121 (42.5%)	5 (71.4%)		NA	
LVI				0.031		0.284
No	234 (80.1%)	231 (81.1%)	3 (42.9%)		1	
Yes	58 (19.9%)	54 (18.9%)	4 (57.1%)		2.608(0.452–15.042)	
Node metastasis				0.006		0.036
No	255 (87.3%)	252 (88.4%)	3 (42.9%)		1	
Yes	37 (12.7%)	33 (11.6%)	4 (57.1%)		6.490 (1.127–37.373)	

EGC, early gastric cancer; SMEGC, synchronous multiple EGC; UT, upper third; MT, mid third; LT, low third; AW, anterior wall; GC, great curvature; PW, posterior wall; LC, lesser curvature; LVI, lymphovascular invasion; NA, not-applicable; VL, vertical location; HL, horizontal location.

However, in patients with undifferentiated EGC (364/680, 53.5%), HER2-positive patients (7/364, 1.9%) showed a significant correlation with LVI (p = 0.031) and presence of LNM (p = 0.006) compared with HER2-negative patients (285/364, 78.3%). Multivariate analysis indicated HER2 overexpression as a good predictive marker of LNM in patients with undifferentiated EGC (odds ratio: 6.490, 95.0% confidence interval: 1.127–37.373, p = 0.036).

### TCGA data analysis of EGC and HER2

**Figure 2:**
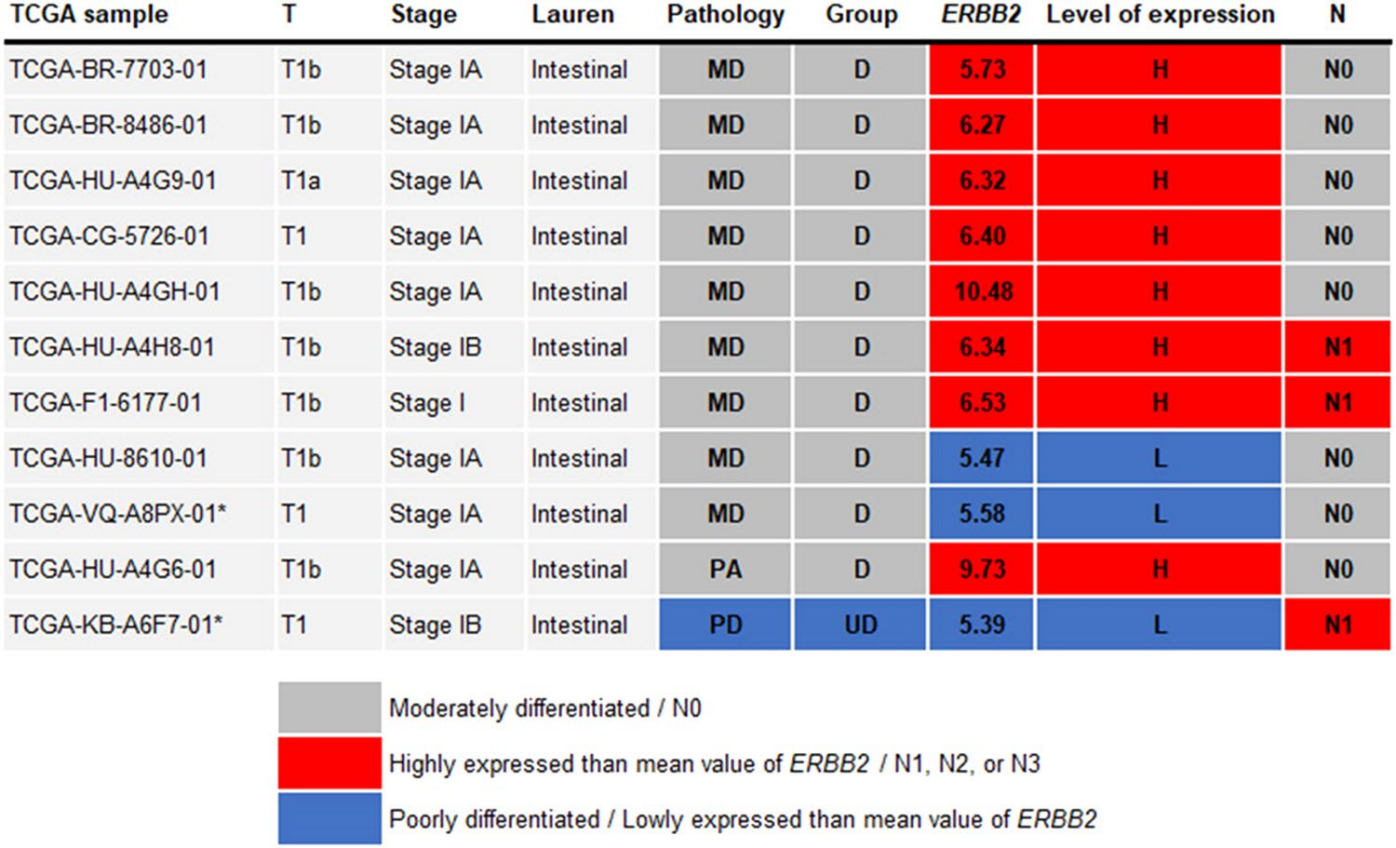
Patients with stage 1 in TNM staging system in the TCGA STAD dataset. We arranged The Cancer Genome Atlas Stomach Adenocarcinoma (TCGA STAD) patient samples that were stage I by T1 tumour stage in the TNM staging system and with available *ERBB2* expression levels. Eleven samples were available and their differentiated statuses were accessed from pathology reports. The *ERBB2* expression level of the poorly differentiated sample, TCGA-HU-A4G6-01, was only slightly higher than the *ERBB2* mean value of whole tumour samples (coloured as red in the *ERBB2* column), and its lymph nodes stage was N0. The *ERBB2* expression of the TCGA-KB-A6F7-01 sample, which was poorly differentiated, was lower than the mean value (coloured as blue in the *ERBB2* column), and its lymph nodes stage was N1 (coloured as red in the N column). #N/A indicates not available. D, differentiated adenocarcinoma; H, high; HER2, Human epidermal growth factor receptor 2; L, low; MD, moderately differentiated cancer; PA, papillary adenocarcinoma; PD, poorly differentiated cancer; UD, undifferentiated adenocarcinoma.

All eleven samples were stage I in the TNM staging system, and all samples were intestinal type in the Lauren classification (Fig. 2). The *ERBB2* expression level of TCGA-HU-A4G6-01 was 9.73, which is higher than the mean value of *ERBB2* (coloured as red), and its lymph nodes were N0 (coloured as grey). TCGA-KB-A6F7-01, which was poorly differentiated, was N1, and its *ERBB2* expression level was lower than the mean value of *ERBB2* (coloured as blue). Two of the remaining nine samples (TCGA-HU-A4H8-01 and TCGA-F1-6177-01), which were identified as differentiated, were N1 in the lymph node stage, and *ERBB2* expression levels were only slightly higher than the mean value of *ERBB2*. Due to the small sample size, a conclusive correlation between *ERBB2* expression level and lymph node stage in poorly differentiated or undifferentiated patients could not be determined with the TCGA STAD dataset.

## Discussion

In the present study, we analysed the correlation of clinical characteristics according to the degree of HER2 expression. Our analysis showed that when HER2 expression increases from ‘negative’ to ‘equivocal’ and further to ‘positive’, the expression patterns were correlated with clinical factors such as age, sex, tumour size, depth of invasion, histology, and lympho-vascular invasion in all EGC patients. Based on this observation, we hypothesised that positive HER2 overexpression would have important clinical implications in tumour aggressiveness.

However, among the total number of patients with EGC, positive HER2 overexpression was not clinically significant compared with negative HER2 overexpression in patients with whole EGC. In the next step, we performed analysis by dividing patients into two groups: differentiated cancer and undifferentiated cancer. There is evidence that HER2 expression differs according to the degree of gastric cancer differentiation and the histologic cancer type^22,23^. Based on these reports, we further performed analysis by subdividing patients into two groups: differentiated-type EGC and undifferentiated-type EGC. Another important reason for dividing into two groups is that differentiated and undifferentiated cancers have different criteria in determining EGC treatment.

In further analysis, in differentiated cancers, HER2 overexpression had no statistical, clinical significance compared with that in negative patients. However, in the analysis of undifferentiated EGC, the HER2 positive group showed more LVI and LNM than the negative group. In multivariate analysis, HER2 overexpression was also identified as a statistically significant risk factor for LNM compared to the HER2 negative group.

Endoscopic or clinical findings alone have limitations in predicting LNM. Therefore, we decided to examine tumour biology because aggressiveness, such as LNM, is closely related to tumour biology, among many molecular mechanisms. In this regard, presence of HER2 overexpression in patients with undifferentiated EGC is an independent risk factor for LNM and may be an important criterion for tailored treatment in EGC patients. In some studies of early gastric neoplasm, HER2 is thought to be involved in the early steps of gastric carcinogenesis, and HER2 overexpression in gastric cancer is a predictor of poor survival^18,23^. In addition, the relationship between HER2 and cancer cell aggressiveness has been reported as follows: amplification of the ERBB2 gene produces an overexpression of HER2 protein that leads to cancer cell survival, growth, and proliferation through the PI3K-AKT and MAPK pathways^20,23,24^. Based on these reports and present results, further studies on the clinical role of HER2 overexpression in EGC should be conducted.

Generally, if HER2 protein expression is 2+ by IHC, it is judged equivocal. Further, fluorescence *in situ* hybridisation (FISH)/silver *in situ* hybridisation (SISH), which is a test for the degree of gene amplification, is additionally performed to confirm HER2 overexpression^25^. In the present study, we did not perform gene amplification in equivocal (score 2+) patients, since no relationship between EGC and HER2 has been confirmed by clinical trials. In addition, the clinical utility of HER2 in EGC is not clear, and therefore, the HER2 expression test in EGC is not conducted routinely.

In studies investigating HER2 expression in stomach cancer using IHC, various definitions of HER2 overexpression were used. Some studies classified equivocal expression as an overexpression group and some as a negative group. Depending on how they are categorised, differences can be observed. Therefore, it is important to define equivocal expression as negative or positive. Thus, various results may be obtained depending on the definition of HER2 positivity^26,27^. In the present study, we performed the final analysis, except equivocal expression, in patients to confirm the clinical significance of HER2 overexpression and to ensure that overexpression had a meaning relative to the negative expression.

In gastric cancer, HER2 overexpression has been reported in approximately 10–30% of cases^23^. In our study, HER2 overexpression was observed in 5.3% of patients with EGC. Futher, the prevalence of HER2 overexpression in out study differs slightly from those of other investigations. The reason can be summarised in two ways. First, HER2 overexpression/amplification, assessed by immunohistochemistry and/or FISH/SISH, has different test methods and different classification criteria. Second, the prevalence of HER2 expression may be difficult to compare directly with those of other studies because the degree of prevalence depends on the histologic type and cancer subtype of the study’s subjects. In this study, HER2 overexpression was significantly different in 9.2% (29/316) of differentiated EGC and 1.9% (7/364) of undifferentiated EGC. This is in line with the results of other studies referring to different HER2 expression according to the degree of gastric cancer differentiation and histologic cancer type^22–24^.

According to the International Controlled Trials (ToGA), HER2 is a target in AGC patients with HER2 overexpression and is reported to be eligible for trastuzumab treatment. The ToGA study was the first clinical trial to demonstrate the survival benefit of AGC patients using a molecular target agent^17^. This study compared the combination of chemotherapy (capecitabine or 5-FU+ cisplatin) with trastuzumab and chemotherapy alone groups, and the addition of trastuzumab significantly improved the median overall survival (13.8 months vs. 11.1 months, *p* = 0.0046). Thus, HER2 was recognised as the first molecular target in gastric cancer, and measurement of HER2 expression status in gastric cancer tissues became very important.

Since the publication of the results of the ToGA trial, it has been recommended that the HER2 test should be performed if a metastatic gastric adenocarcinoma is present or suspected^25,28^. If HER2 protein expression is 2+ by IHC, it is judged equivocal. Further, FISH/SISH, which is a test for the degree of gene amplification, is additionally conducted to confirm HER2 overexpression^25,29^. In this way, AGC is used to confirm HER2 expression level. However, since EGC has not been studied yet and its clinical utility has not been confirmed, the HER2 expression test has not been performed in many cases.

According to the present results, HER2 is valuable molecular marker in EGC and plays an important clinical role. When EGC with HER2 overexpression is diagnosed, it should be considered an important warning in undifferentiated EGC. Although imaging studies, such as computed tomography and endoscopic ultrasonography, do not detect LNM, more aggressive testing or treatment should be required. For example, diagnostic tests based on invasive interventions, such as laparoscopic biopsy or sentinel node biopsy, should be actively considered. In addition, it may be necessary to choose surgical operation rather than endoscopic treatment. It seems that HER2 overexpression may play an important role in EGC as in AGC. Furthermore, we would suggest that HER2 expression test should be performed in all EGC patients in the future.

There is little research on the clinical implications of HER2 overexpression in EGC. Therefore, we conducted additional analysis on TCGA data. In TCGA data, there was no correlation between HER2 overexpression and LNM in the differentiated group. Although ERBB2 gene expression and HER2 protein expression identified by IHC do not have the same status, this result is in the same context as our retrospective data results. We also analysed the undifferentiated group, but there was only one sample of undifferentiated cancer, which was not suitable for a meaningful analysis. The present study reports the clinical utility of the HER2 test for EGC for the first time. Even the TCGA data are insufficient to confirm the usefulness of HER2 in EGC. It is thought that this will be the starting point of studies that establish the role of HER2 in EGC in the future.

However, there are limitations to this study. First, it is a retrospective study from a single centre. Second, the statistical power is weak because of the small number of events of HER2 expression in undifferentiated cancer. Despite these weak points, HER2 overexpression may be clinically very useful. This is because HER2 expression test as a tool for predicting LNM in undifferentiated carcinoma has 98.82% specificity, 88.42% negative predictive value, and 87.67% accuracy. Although the sensitivity is only 10.81%, the high specificity of the HER2 test is very important, suggesting that in EGC patients in which the pathological outcome from endoscopic resection is undifferentiated cancer and HER2 expression is negative, there would be little LNM. Thus, it can be assumed that the patient does not need additional surgery or treatment.

In conclusion, HER2 overexpression is an independent risk factor for LNM compared with negative expression in undifferentiated EGC. Therefore, the possibility that HER2 may have clinical significance as in AGC should be considered in EGC. Even if the imaging study shows no LNM, clinicians should be cautious if there is presence of HER2 overexpression in undifferentiated EGC. However, the results of this study are not directly applicable for testing HER2 in all EGC patients. If further studies confirm the usefulness of HER2 in EGC, the criteria for HER2 testing in EGC, as in AGC, will be clear.

## Methods

### Study population

**Figure 3:**
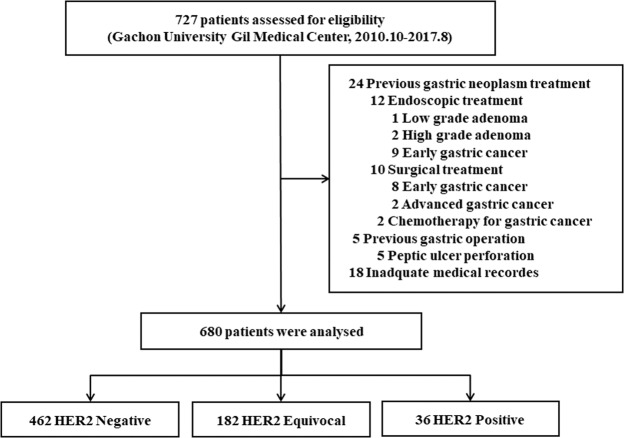
Flow chart of patient selection in this study. HER2, Human epidermal growth factor receptor 2.

We retrospectively reviewed the medical records of patients who underwent surgery for EGC between October 2010 and August 2017 at the Gachon University Gil Medical Center. Of the 727 patients, 24 who were treated for previous gastric neoplasms, 5 who underwent gastric operation for ulcer perforation, and 18 whose medical records were insufficient were excluded. Of 24 cases mentioned above, 17 had previously received EGC treatment. The study included patients who were initially diagnosed with EGC, so those 17 EGC-treated patients were also excluded. Finally, 680 patients were included in the analysis (Fig. 3). The study was conducted under approval of the institutional review board of the Gachon University Gil Medical Center (IRB No. GAIRB2018-411), in accordance with the provisions of the Declaration of Helsinki and Good Clinical Practice guidelines. Informed consents with adequate explanation for the diagnostic test and treatment modality were obtained from each patient.

### Procedure and definitions

The attending surgeon chose the sort of surgery and decided the extent of lymphadenectomy relying upon the location of the cancer and general performance of the patient. Whenever conditions permitted, curative intent surgery with R0 resection and D2 lymph node dissection was performed.

As indicated by the Japanese Gastric Cancer Association criteria, the gastric cancer type was characterised based on location (longitudinal or horizontal), histological features, and macroscopic type^30^. Anatomically, the stomach consists of three parts along its longitudinal axis: upper third, middle third, and lower third. On a horizontal plane, the circumference of the stomach is separated into four parts: anterior wall, lesser curvature, posterior wall, and greater curvature. During the study, the tumour sizes were measured by the longest diameter of the lesion in the surgical specimen. In terms of macroscopic classification, tumours were classified into three types as follows: elevated (types 0-I, 0-IIa, 0-I + IIa, 0-IIa + IIb, 0-IIa + IIc); flat (type 0-IIb); and depressed (types 0-IIc, 0-III, 0-IIc + IIa, and 0-III + IIa). Histologically, tumours were characterised into two distinct types, which were differentiated and undifferentiated. Differentiated type was classified into papillary adenocarcinoma or well and moderately differentiated tubular adenocarcinoma. Undifferentiated type consisted of three subtypes, which were poorly differentiated tubular adenocarcinoma, signet-ring cell carcinoma, or mucinous adenocarcinoma^8^.

HER2 expression determined by IHC was analysed in EGC and was classified as negative (0 and 1+), equivocal (2+), or positive [overexpression (3+)]^25,31^. The definition for each was as follows; Negative (0): No response to staining, or membrane responds to staining in <10% of tumour cells (Cytoplasmic staining was not included in the scoring); Negative (1+): ≥10% of tumour cells with very faint staining or barely recognisable staining; Equivocal (2+): refers to the case of weak or moderate complete staining on the basolateral and lateral sides in ≥10% of tumour cells (Tangentially cut cells in columnar cells tended to show a complete membrane staining pattern); Positive(3+): ≥10% of tumour cells show a strong complete staining reaction in the basolateral and lateral membranes. It should be taken into account that some of the cells that appeared to show a complete membrane staining pattern were often tangentially cut columnar cells.

### TCGA STAD dataset

We downloaded The Cancer Genome Atlas Stomach Adenocarcinoma (TCGA STAD) clinical dataset, version 2016-04-27 from the University of California at Santa Cruz Xena Browser (xena.ucsc.edu/) (last accessed on 2018-04-04)^32,33^. Eleven samples were identified with our criteria with a pathological tumour of ‘T1’, ‘T1a’, and/or ‘T1b’, sample type as ‘solid tumour’, pathological lymph nodes stage not ‘NX’, and available messenger RNA expression of HER2 (*ERBB2*) in the TCGA transcriptome dataset. We identified the differentiation status of each sample with the ‘Pathology Report’ from cBio Portal^34^. The TCGA data for Oesophagus-Stomach Cancers were used^35^. *ERBB2* expression levels were encoded into two values, ‘high’ and ‘low’ according to the *ERBB2* mean value of TCGA STAD tumour samples (the mean value was 5.66, and the unit was log2 (RPKM + 1)).

### Statistical analysis

The statistical program used in this study was SPSS 22.0 software (IBM SPSS Statistics, IBM Corporation, Armonk, NY, USA) for MS Windows®. Continuous variables are expressed as mean ± standard deviation, and interval variables are expressed as absolute numbers and percentages. We used a linear-by-linear association test (chi-square test for trend) to investigate the interrelationship between the expression intensity of HER2 protein and clinicopathological characteristics in patients with EGC. After excluding equivocal expression of the protein, the Pearson 2 or Fisher’s exact test for univariate analysis was performed to determine differences in the characteristics of patients with HER2 protein expression in comparison with those without HER2 protein expression. Multivariate analysis by logistic regression was performed using the statistically significant variables found in the univariate analysis. Two-tailed P-values of 0.05 or less were considered statistically significant.

## Data Availability

The data sets generated and/or analyzed during the current study are available from the corresponding author on reasonable request.

## References

[1] Carlomagno N (2017). Diagnostic, Predictive, Prognostic, and Therapeutic Molecular Biomarkers in Third Millennium: A Breakthrough in Gastric Cancer. BioMed research international.

[2] Jung KW (2017). Cancer Statistics in Korea: Incidence, Mortality, Survival, and Prevalence in 2014. Cancer Res. Treat.

[3] Jun JK (2017). Effectiveness of the Korean National Cancer Screening Program in Reducing Gastric Cancer Mortality. Gastroenterology.

[4] Kim YG (2014). Effects of screening on gastric cancer management: comparative analysis of the results in 2006 and in 2011. J. Gastric. Cancer.

[5] Hosokawa O (2008). Decreased death from gastric cancer by endoscopic screening: association with a population-based cancer registry. Scand J. Gastroenterol..

[6] Nam SY (2009). Effect of repeated endoscopic screening on the incidence and treatment of gastric cancer in health screenees. Eur. J. Gastroenterol. Hepatol..

[7] Roviello, F. *et al*. Number of lymph node metastases and its prognostic significance in early gastric cancer: a multicenter Italian study. *J. Surg. Oncol.* **94**, 275–280; discussion 274, 10.1002/jso.20566 (2006). 10.1002/jso.20566 16917863

[8] Japanese Gastric Cancer A (2017). Japanese gastric cancer treatment guidelines 2014 (ver. 4). Gastric Cancer.

[9] Gotoda T (2000). Incidence of lymph node metastasis from early gastric cancer: estimation with a large number of cases at two large centers. Gastric Cancer.

[10] Kwee RM, Kwee TC (2008). Predicting lymph node status in early gastric cancer. Gastric Cancer.

[11] Tanabe S (2017). Long-term outcomes of endoscopic submucosal dissection for early gastric cancer: a multicenter collaborative study. Gastric Cancer.

[12] Horiuchi Y (2019). Pretreatment diagnosis factors associated with overtreatment with surgery in patients with differentiated-type early gastric cancer. Scientific reports.

[13] Abdelfatah MM (2018). The incidence of lymph node metastasis in early gastric cancer according to the expanded criteria in comparison with the absolute criteria of the Japanese Gastric Cancer Association: a systematic review of the literature and meta-analysis. Gastrointest Endosc..

[14] Ryu ES (2019). Sex-specific differences in risk factors of lymph node metastasis in patients with early gastric cancer. PloS one.

[15] Tae CH (2015). Is endoscopic resection an alternative to surgery for early low-risk submucosal gastric cancers: analysis of a large surgical database. Surg. Endosc..

[16] Jo MJ (2015). Biopathologic features and clinical significance of micrometatasis in the lymph node of early gastric cancer. World J. Gastroenterol..

[17] Bang YJ (2010). Trastuzumab in combination with chemotherapy versus chemotherapy alone for treatment of HER2-positive advanced gastric or gastro-oesophageal junction cancer (ToGA): a phase 3, open-label, randomised controlled trial. Lancet.

[18] Yan Y (2015). HER2/neu over-expression predicts poor outcome in early gastric cancer without lymph node metastasis. Clinics and research in hepatology and gastroenterology.

[19] Liang JW, Zhang JJ, Zhang T, Zheng ZC (2014). Clinicopathological and prognostic significance of HER2 overexpression in gastric cancer: a meta-analysis of the literature. Tumour biology: the journal of the International Society for Oncodevelopmental Biology and Medicine.

[20] Klapper LN, Kirschbaum MH, Sela M, Yarden Y (2000). Biochemical and clinical implications of the ErbB/HER signaling network of growth factor receptors. Adv. Cancer Res..

[21] Fassan M (2012). Early HER2 dysregulation in gastric and oesophageal carcinogenesis. Histopathology.

[22] Gravalos C, Jimeno A (2008). HER2 in gastric cancer: a new prognostic factor and a novel therapeutic target. Annals of oncology: official journal of the European Society for Medical Oncology/ESMO.

[23] Ieni A, Barresi V, Rigoli L, Caruso RA, Tuccari G (2015). HER2 Status in Premalignant, Early, and Advanced Neoplastic Lesions of the Stomach. Disease markers.

[24] Gallardo A (2012). Increased signalling of EGFR and IGF1R, and deregulation of PTEN/PI3K/Akt pathway are related with trastuzumab resistance in HER2 breast carcinomas. Br. J. Cancer.

[25] Bartley AN, Washington MK, Ismaila N, Ajani JA (2017). HER2 Testing and Clinical Decision Making in Gastroesophageal Adenocarcinoma: Guideline Summary From the College of American Pathologists, American Society for Clinical Pathology, and American Society of Clinical Oncology. Journal of oncology practice.

[26] Creemers A (2017). Discordance in HER2 Status in Gastro-esophageal Adenocarcinomas: A Systematic Review and Meta-analysis. Scientific reports.

[27] Wong N (2018). HER2 testing of gastro-oesophageal adenocarcinoma: a commentary and guidance document from the Association of Clinical Pathologists Molecular Pathology and Diagnostics Committee. J. Clin. Pathol..

[28] Jimenez-Fonseca P (2017). Prognostic significance of performing universal HER2 testing in cases of advanced gastric cancer. Gastric Cancer.

[29] Zakrzewski F (2019). Automated detection of the HER2 gene amplification status in Fluorescence *in situ* hybridization images for the diagnostics of cancer tissues. Scientific reports.

[30] Japanese Gastric Cancer A (2011). Japanese classification of gastric carcinoma: 3rd English edition. Gastric Cancer.

[31] Hofmann M (2008). Assessment of a HER2 scoring system for gastric cancer: results from a validation study. Histopathology.

[32] University of California at Santa Cruz Xena Browser, The Cancer Genome Atlas Stomach Adenocarcinoma (TCGA STAD) clinical dataset, version 2016-04-27, http://xena.ucsc.edu/ (last accessed on 2018-04-04).

[33] Cancer Genome Atlas Research, N. Comprehensive molecular characterization of gastric adenocarcinoma. *Nature* **513**, 202–209, 10.1038/nature13480 (2014). 10.1038/nature13480 PMC4170219 25079317

[34] Gao J (2013). Integrative analysis of complex cancer genomics and clinical profiles using the cBioPortal. Science signaling.

[35] Cancer Genome Atlas Research, N. *et al*. Integrated genomic characterization of oesophageal carcinoma. *Nature* **541**, 169–175, 10.1038/nature20805 (2017). 10.1038/nature20805 PMC5651175 28052061

